# Trend and Causes of Overweight and Obesity among Pre-School Children in Kuwait

**DOI:** 10.3390/children8060524

**Published:** 2021-06-19

**Authors:** Nawal Alqaoud, Ayoub Al-Jawaldeh, Fahima Al-Anazi, Monica Subhakaran, Radhouene Doggui

**Affiliations:** 1 Food and Nutrition Administration, Ministry of Health, Kuwait City 13001, Kuwait; dnmalq@gmail.com (N.A.); qiwiq8@hotmail.com (F.A.-A.); monicakaran@gmail.com (M.S.); 2 Regional Office for the Eastern Mediterranean (EMRO), World Health Organization (WHO), Cairo 11371, Egypt; aljawaldeha@who.int; 3 Department of Family Medicine, Université de Sherbrooke, Sherbrooke, QC J1K 2R1, Canada; 4 Centre de Formation Médicale du Nouveau–Brunswick, Moncton, NB E1A 3E9, Canada

**Keywords:** obesity, body mass index, sugary and sweetened beverages, breakfast skipping, sedentary behavior, childhood, Eastern Mediterranean region

## Abstract

Identifying life risk factors of obesity early will help inform policymakers to design evidence-based interventions. The following study aims to assess the trend of overweight and obesity over four years among pre-school Kuwait children, and to examine their association with breakfast skipping (BF), sugary and sweetened beverage (SSB) consumption, and screen time. Children aged 2–5 years (*n* = 5304) were selected from 2016 to 2019 national surveys. Overweight and obesity were defined according to the World Health Organization references. The children’s mothers were asked about the BF of their children the day of the survey, their frequency of SSB consumption, and their weekly screen time use. Logistic regression was used to identify the risk factors associated with overweight/obesity. No significant decline (*p* values ≥ 0.12) was found for both overweight and obesity. Contrastingly, BF skipping, SSB consumption, and screen time declined (*p* < 0.0001). The BF skippers were found to have a 31% lower risk of being overweight. Daily TV watching, for 2–3 h, increases the odds of obesity by 5.6-fold. Our findings are encouraging regarding the decline in risky behaviours over time. However, more effort should be made both at the micro- and macro-level for a sustainable reduction in overweight and obesity.

## 1. Introduction

Over the past decades, overweight and obesity reached an alarming rate globally [1]. The worldwide prevalence of obesity increased by 5.9% from 1975 to 2016 [2]. Early life obesity was identified as a long-life determinant of morbidity and chronic diseases (e.g., metabolic risk factors, cardiovascular diseases, and cancers) during adolescence and later on in adulthood [3,4]. Even more, acquiring healthy lifestyles during the early years of life, specifically the first five years, is a determinant for the children’s growth and neurocognitive development [5].

Similarly to other Gulf countries, Kuwait has undergone a rapid economic and social transition that significantly impacted the population’s lifestyle [6]. There is evidence that the Kuwait population is experiencing a nutrition transition, portrayed by the emergence of overweight and obesity [7,8]. A recent national cross-sectional study showed that overweight and obesity among school-age children (5–19 years) reached 20.2% and 28.4% in 2019, respectively. These proportions are within the highest ones in the Eastern Mediterranean region.

At the individual level, physiological, environmental and behavioural factors overlap to cause an excess of adiposity [9]. Of the behavioural factors, poor eating habits, such as breakfast skipping [10,11], and the overconsumption of sugar and sweetened beverages [12], as well as a sedentary lifestyle, such as screen overuse [13], have been associated with childhood obesity and connected to the nutrition transition. Indeed, a meta-analysis conducted by Ardeshirlarijani et al. [11] showed an increased risk of overweight among children and adolescents skipping breakfast (odds ratio 1.44 with 95% CI (1.31–1.59)). However, a subgroup analysis showed that only cross-sectional studies reported a significant association, while longitudinal studies did not support this relationship [11]. According to the available data, breakfast skipping seems frequent among older children and adolescents, with more than 50% of skippers [14,15]. However, this eating habit of skipping breakfast may track back from childhood. Stated differently, breakfast skipping may have been acquired during childhood [16]. The World Health Organization (WHO) has recommended restricting free sugar intake, especially derived from sugary and sweetened beverages [17]. A meta-analysis including fifteen studies, conducted among children and adolescents, showed that the increase in sugary and sweetened beverage consumption by a 12 oz serving/d was associated with an increase in body weight, by +0.07 [18]. At the time of the study, data on sugary and sweetened beverage consumption among Kuwait children under 5 years old are unavailable. However, a study conducted among school-age children reported that 43% had more than one soft drink per day [19]. Next to the eating behaviours, screen use for two hours and more was found to increase overweight and obesity risk [13]. Accordingly, an assessment realized among 435 Kuwaiti adolescents revealed that 79% had more than one hour per day of screen use [20]. Similar findings were reported among Saudi school-age children [21].

To date, little is known about these correlates of overweight and obesity among children under five years, especially in Kuwait as an example of Gulf countries and as a part of the Eastern Mediterranean region. Therefore, the following study aims to examine (1) the trend of overweight and obesity over four years (2016–2019); (2) the association between breakfast skipping, sugary and sweetened beverages, and screen use time with overweight and obesity among Kuwait children aged from 2 to 5 years.

## 2. Methods

### 2.1. Kuwait Nutrition Surveillance System and Subjects

The State of Kuwait established nutrition surveillance system in 1995 and has been it running successfully for more than 20 years. The Kuwait nutrition surveillance system (KNSS) has been designated to collect, analyze, and disseminate surveillance data to guide public health policy and action. The data are collected only among Kuwaiti citizens using standardized data collection forms through personal interviews conducted by trained field agents. The overall objective of KNSS is to provide regular and updated information on the nutritional status of the Kuwaiti population (children and adults) and the influencing factors. Because the national representative survey is conducted yearly, the KNSS allows assessing nutritional status trends over time. Children aged 24–60 months were recruited from eight health centres in all the six Kuwait governorates at the time of their vaccination from January 2015 to December 2018. The free vaccination in Kuwait and the high coverage (almost 100%) allow the generation and access to a representative sample of Kuwaiti children [8]. The KNSS accounts for a pre-established list of health centres designated to be sentinel sites for nutritional surveillance in all governorates. No sampling method was used as every mother or child guardian attending the vaccination centres was invited to participate in data collection, and only less than 2% refused to participate. For the purpose of this repeated national cross-sectional study among pre-school children, the interview was conducted with mothers or children’s guardians.

### 2.2. Anthropometric Assessment

Trained dieticians collected all anthropometric data and measurements. Standing height was measured to the nearest 0.1 cm using an electronic stadiometer (SECA model 220); weight was measured to the nearest 0.1 kg on a calibrated scale (SECA Alpha, medical scales and measurement systems, Hamburg, Germany). Concerning children <5.09 WHO growth standard was applied, as follows: overweight was defined as body mass index (BMI)-for-age ≥+2 z and obesity ≥ +3 z, respectively [22]. For children aged ≥5.09 years, we used WHO of 2007, as follows: overweight was defined as BMI-for-age >+1 z score and obesity as >+2 z score [23]. BMI-for-age z-scores were calculated using WHO Anthro Plus software [24].

### 2.3. Eating Behaviours

During the day of the survey, data on breakfast consumption were collected by addressing the following question to the mother: ‘when does your child have breakfast?’ The mother was asked to select one of the following responses: before 7 am, 7–9 am, 9–11 am and after 11 am. Participants having their breakfast after 11 am were identified as skippers.

The weekly frequency of sugary and sweetened beverages consumption was assessed through two questions, namely, ‘How many times per week does your child consume carbonated drinks?’ and ‘How many times per week does your child consume non-fresh drinks (industrialized juices)?’. For these questions, response options included the following: ‘None’, ‘one per week’, ‘two times per week’, ‘three times per week’, ‘four times per week’, ‘five times per week’, ‘five times per week’, and ‘six times per week and more’.

### 2.4. Screen Time

Four questions were used to assess screen time ‘How long does the child watch television during the weekend?’, ‘How long does the child watch television daily?’, ‘How long does the child play computer games during the weekend?’, and ‘How long does the child play computer games daily?’. For these questions, response options included ‘less than 2 h’, ‘2–3 h’ and ‘more than 3 h’.

### 2.5. Covariates

Data relative to children’s birth date, gender and living region were also collected during the survey day.

### 2.6. Data Management and Statistical Analysis

Stata 16 (Stata Corporation, College Station, TX, USA, 2019) was used for data management and statistical analyses. Results were presented as estimates and standard error or a 0.95 confidence interval. The logistic regression was used to assess crude and adjusted associations of overweight and obesity with breakfast skipping, sweetened beverage consumption and screen time. Models’ adjustment was made for the living region, gender and age. Linear regression was used to examine BMI-for-age z score adjusted association with breakfast skipping, sweetened beverage consumption and screen time. An interaction term for sugary and sweetened beverages with daily time screen (TV and computer) was tested. Still, it was not retained for further analysis as it did not reach significance. Associations of overweight and obesity with gender, age, residence, and year of study were assessed using the Chi-square test. Orthogonal polynomial contrasts were used to test BMI-for-age z score across study period for linear trends.

## 3. Results

### 3.1. Sociodemographic Characteristics

The total study includes 4400 participants, of which 49.5% were girls. The gender distribution was similar (*p* = 0.058) across the study period (2016–2019). The mean average among all the participants was 3.22 ± 0.01 years and *Jahra* governorate was the most represented one (*p* < 0.0001), with 23.3%. The details by year of study are provided in Table 1.

### 3.2. Prevalence of Overweight and Obesity

#### 3.2.1. BMI-for-age z score 

For children under 5.09 years, the indicator increased consistently among boys, from 0.0.38 ± 0.05 in 2016 to 0.56 ± 0.06 in 2019 (*p* = 0.023). A similar trend was found for girls (0.42 ± 0.05 in 2016 vs. 0.54 ± 0.06 in 2019), but it did not reach significance (*p* = 0.094). For the children ≥5.09, a negative, but not significant, trend was found for both boys (0.11 ± 0.42 in 2016 vs. −0.23 ± 0.37 in 2019; *p* = 0.50) and girls (0.50 ± 0.34 in 2016 vs. −0.01 ± 0.24 in 2019; *p* = 0.21).

#### 3.2.2. Overweight

**Figure 1:**
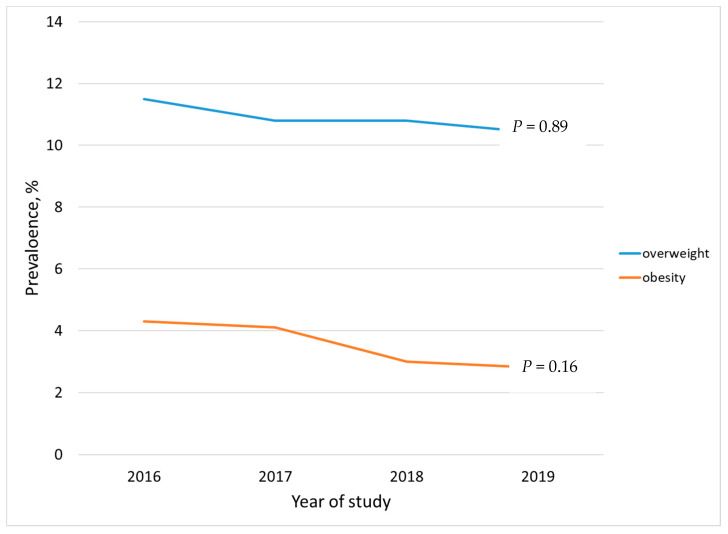
Trend of overweight and obesity prevalence among Kuwait children under 5 years (2016–2019). Figure caption: Chi-squared test was used to assess the prevalence change over time.

The overweight prevalence (Table 2) seems to decline but did not reach the significance level (Figure 1). Across all the samples, the overweight prevalence was 10.9%, and increased significantly in parallel with age (*p* < 0.0001), with a steep rise at 5 years. By year, the same trend was found only during 2017 (9.2% at 2 years vs. 33.3% at 5 years). Overweight was evenly distributed across gender (*p* = 0.42) and governorates (*p* = 0.17) among all the samples. An evenly overweight distribution was found across the governorates during 2016 and 2018. In 2016, the highest overweight prevalence was found in the *Hawali* governorate (*p* = 0.038), while during 2018 it was in the *Ahmadi* governorate (*p* = 0.019).

#### 3.2.3. Obesity

The prevalence of obesity (Table 2) did not change over the four years of study (Figure 1). Overall, the obesity prevalence affected 3.7% of the children and was positively associated with age (*p* < 0.0001). In 2017, the obesity prevalence ranged between 2.7% among children aged 2 years old, to 19.3% among those aged 5 years old. A significant difference was depicted across the governorates (*p* = 0.039) considering the whole sample.

### 3.3. Level of Breakfast Skipping, Sugar and Sweetened Beverages Consumption and Time of Screen Use

#### 3.3.1. Breakfast skipping

Almost a quarter of the children were breakfast skippers (Table 3). We found a significant declining trend of breakfast skipping from 2016 to 2019 (*p* < 0.0001). *Sugary and sweetened beverages:* Table 3 showed that the frequency of sugary and sweetened beverage consumption decreased significantly (coef. = −0.29, 95% CI (−0.49–−0.10)) from 2016 (5.9 times per week) to 2019 (5.6 times per week).

#### 3.3.2. Time of screen use

Irrespective of the used device (computer or TV), the time of screen use decreased significantly (*p* < 0.0001).

### 3.4. Individual-Level Association of Breakfast Skipping, Sugary and Sweetened Beverages and Time of Screen Use with Overweight, Obesity and BMI-for-Age z Score

The logistic regression (Table 4) showed that breakfast skipping reduces the risk of overweight significantly, by 32%. Daily watching of the TV for 2 to 3 h increases the risk of being overweight by 2-fold, and the risk of being obese by 5.6-fold, respectively. Daily computer use during the week (crude OR = 1.68 95% (1.16–2.43)) and during the weekend (crude OR = 1.55 with 95% (1.07–2.25)) for 2 to 3 h were found to be a risk factor of obesity, but these associations did not stand in the adjusted analysis. The linear regression revealed that the increase in sugary and sweetened beverage intake by one time per week is equivalent to an increase in BMI-for-age by +0.02 unit (*p* = 0.036).

## 4. Discussion

Based on national data collected through the Kuwait nutritional survey system, we analyzed the prevalence of overweight and obesity trends among children age from 2 to 5 years, over a period of four years (2016–2019). This is the first study in the Eastern Mediterranean region to assess the association between overweight and obesity with breakfast skipping, sugary and sweetened beverage consumption, and screen use simultaneously among pre-school children. Overweight was found to prevail among a tenth of the children. Furthermore, daily watching of the TV, and sugar and sweetened beverage consumption were associated with overweight and an increased BMI-for-age z score, respectively.

### 4.1. Overweight and Obesity

The overweight and obesity prevalence remained stable over the four years of the study. Only 10% of the children were overweight, while obesity was below 5%. In comparison with neighbourhood countries, we found a higher overweight prevalence than in Oman (4.4%); and lower than in the Kingdom of Saudi Arabia (15.7%) [25], in Egypt (17.1%) [26], and also in Tunisia (17.2%) [27].

### 4.2. Breakfast Skipping

Despite the decrease in breakfast skipping, by 2% over the four years of study, the frequency deemed elevated as a quarter of children did not eat breakfast on the day of the survey. In our study, breakfast skipping prevalence was similar to the prevalence reported in the United States [28], but higher than the reported data in other countries [10,29,30,31]. Indeed, a Chinese study, conducted among 1269 children aged six years, found that only 5.7% of the children reported having breakfast less than four days per week [30]. Another study realized among Canadian pre-school children (n = 2103) found that 10% of them were classified as skippers [29]. Breakfast skipping was found to be protective against overweight in our study; a simple explanation could be that skippers tend to have a lower daily energy intake. The available studies conducted among children and adolescents report a positive association between overweight and breakfast skipping [11]. Consistently, the few studies conducted among pre-school children are showing non-congruent patterns of association. Breakfast skippers have been reported to be at a two-fold higher risk of overweight in a Canadian study [10]. An Australian study, carried out among children aged 2–16 years old, showed that the prevalence of overweight was more important among breakfast skippers vs. consumers [32]. A second longitudinal Australian study showed a positive association between overweight and breakfast skipping (2–3 years, OR = 2.4; 4–5 years, OR = 2.32; *p* < 0.05) [33]. Contrastingly, another longitudinal study was conducted among Dutch children at 2 years old and 5 years old, and overweight was not associated with breakfast skipping [31]. The lack of a standard definition might explain the inconsistent association of breakfast status and an excess of adiposity for ‘breakfast skipping’. As well as this, the difference in study design (cross-sectionally vs. longitudinally [11]) or the considered confounders, such as socio-economic level or mother’s education levels, proved to be determinants of child nutritional status [9] and breakfast [34].

### 4.3. Sugary and Sweetened Beverages

To our knowledge, our study is the first conducted study in the Eastern Mediterranean region studying the association between sugary and sweetened beverage consumption with overweight among pre-school children. A modest, but significant, association was found between the frequency of sugary and sweetened beverage consumption and BMI-for-age z score. In a study carried out in Florida (United States), among children aged between 3 and 4.9 years, overweight status was a predictor of increased sugary and sweetened beverage intake by 2.74 oz per day [35]. A Canadian study, conducted by Danyliw and al. [36], found no association between BMI and beverage clusters (fruit drink, soft drink, and fruit juice). Another conducted study in the United States found a significant difference in overweight prevalence across beverage clusters. However, after adjustment to confounders in regression analysis, the association did not stand [37]. Another study by Vinke and al. [38] found that children belonging to the highest quartile of sugary and sweetened beverage consumption, compared to the lowest one, are at a three-fold higher risk of overweight. Similar findings were found elsewhere [39].

Next to the association of BMI-for-age z score with sugary and sweetened beverages, Kuwait pre-school children were found to have a high frequency of sugary and sweetened beverages ≈ 6 times per week. Nonetheless, the frequency of consumption declined, with a more pronounced decrease for soft drinks. The substantial progress made might be attributed to a national policy aiming to control chronic disease, which includes an action dedicated to limiting non-alcoholic beverage consumption among children [40].

### 4.4. Time of Screen Use

A quarter of the children watched TV and played on the computer daily for more than 2 h. The used question responses of our questionnaire do not allow the identification of participants that do not meet the recommended one hour per day [41]. However, low adherence to the recommendation affects at least 32% of children (cumulating ≥2 h of screen use daily). Overuse of screens among pre-school children is not exclusive to Kuwait children; in Canada, for example, 85% of pre-school children do not comply with the recommendation. In the United States, children aged between 8 months to 8 years old were found to be exposed to four hours of background TV [42]. There is a need for public health intervention to raise parents’ awareness that early exposure to screen overuse increases the odds of excessive use later [43].

### 4.5. Recommendations

Breakfast is the ‘motor of the day’ and contributes significantly to daily nutrient intake. Even if breakfast skipping was associated with a lower risk of overweight, the promotion of healthy daily breakfast consumption is recommended. The promotion goes through raising parents’ awareness about the possible health outcomes related to breakfast skipping (e.g., lower vitamins and trace elements intake). The results provide solid evidence of the importance of discouraging parents from offering sugary and sweetened beverages to their children. The use of the front-of-pack label is recommended, as done in some other countries in the EMR region (e.g., Kingdom of Saudi Arabia and Morocco) [44]. Specifically, warning labels regarding the harms of added sugars could help to encourage parents to make healthier choices for food. This is more important because parents exposed to a warning label tend to pass over-sweetened and sugary beverages [45]. Finally, there is a need to decline screen use by informing parents, through school programs, about the limited number of hours that their children should spend in front of a screen and the cognitive impairments of overuse. At the same time, the introduction of healthy, enjoyable activities is essential for displacing screen time and for social acceptance as an alternative solution [46].

### 4.6. Strengths and Limitations

This study has several strengths. This study includes a large population, a national representative of Kuwait children (2–5 years), and covers four successive years. Also, all anthropometric measures were measured and not self-reported. However, an important limitation of this study is that several confounders were not considered, such as the mother’s education level, physical activity level, and energy intake level. Furthermore, we used food frequency assessment of sugary and sweetened beverage consumption as well as breakfast eating, which may hide the effective quantity of consumed beverages. Also, as data relative to breakfast skipping were collected for one day, we do not account for the intra-individual variability. Furthermore, data were collected retrospectively among mothers, which is a source of recall bias. Also, a non-validated questionnaire was used to collect this dietary data. Lastly, measurements of waist circumference, triceps fold, and level of physical activity were not collected in the following study.

## 5. Conclusions

To conclude, this study underlines the impact of nutrition transition among Kuwait pre-school children, translated by a high prevalence of overweight (almost a tenth), high sugary and sweetened beverage consumption, high breakfast skipping rate, as well as overuse of screens, which is a marker of a sedentary lifestyle. Although overweight and obesity prevalence did not decline over time, there is a tremendous reduction in sugary and sweetened beverages (i.e., soft drinks by two-fold) as well as breakfast skipping over four years. Furthermore, there is a decline in screen use time, which might be a marker of an increased level of children’s physical activity. This study added more to the debate on eating behaviours and the association with an excess of adiposity among pre-school children, for whom the data are deemed scarce in the Eastern Mediterranean region.

## Figures and Tables

**Table 1:** Socio-demographic characteristics.

	2016	2017	2018	2019	*p* Value
**N**	1184	1378	832	1006	
**%**	26.9	31.3	18.9	22.9	
**Age**	3.25 ± 0.03 ^3^	3.32 ± 0.02	3.13 ± 0.03	3.20 ± 0.03	0.007 ^1^
**Girls (%)**	51.7	47.0	50.8	51.4	0.058 ^2^
**Governorate**					
Capital	13.8	16.3	10.9	12.2	<0.0001 ^2^
Hawali	6.1	10.9	18.3	10.4	
Farwanyia	12.0	21.7	21.4	25.3	
Ahmadi	19.6	16.0	11.3	21.8	
Jahra	32.3	21.7	19.1	25.2	
Mubaral Al Kabeer	16.2	13.5	19.0	5.5	

^1^*p* value for the comparison of the means across the period of study by linear regression. ^2^ *p* value issued by the Chi-squared test. ^3^ Mean ± standard error of mean.

**Table 2:** By year, overweight and obesity prevalence among Kuwait children under 5 years.

	2016	2017	2018	2019	*p* Value (Overall)
N	1184	1378	832	1006	
	**Overweight**				
**Age**	*p* ^1^ = 0.17	*p*^1^ < 0.0001	*p*^1^ = 0.16	*p*^1^ = 0.87	<0.0001
2 years	12.6	9.2	9.4	10.8	
3 years	10.2	10.5	12.0	9.7	
4 years	10.5	9.2	8.24	10.4	
5 years	20.5	33.3	22.7	13.5	
**Gender**	*p*^1^ = 0.48	*p*^1^ = 0.48	*p*^1^ = 0.51	*p*^1^ = 0.35	0.53
Boys	10.8	10.3	10.1	11.3	
Girls	12.2	11.5	11.5	9.5	
**Governorate**	*p*^1^ = 0.038	*p*^1^ = 0.99	*p*^1^ = 0.011	*p*^1^ = 0.37	0.032
Capital	8.6	10.7	14.3	8.9	
Hawali	20.0	10.1	11.3	12.8	
Farwanyia	6.3	11.0	3.9	8.4	
Ahmadi	14.2	10.6	16.1	10.5	
Jahra	11.8	10.7	14.3	13.2	
Mubaral Al Kabeer	11.0	11.8	9.5	5.6	
	**Obesity**				
**Age**	*p*^1^ = 0.13	*p*^1^ < 0.0001	*p*^1^ = 0.019	*p*^1^ = 0.99	
2 years	3.7	2.7	2.1	2.9	<0.0001
3 years	4.3	3.7	3.5	2.7	
4 years	4.1	4.1	2.4	3.0	
5 years	11.4	19.3	13.7	2.7	
**Gender**	*p*^1^ = 0.71	*p*^1^ = 0.64	*p*^1^ = 0.34	*p*^1^ = 0.	
Boys	4.6	3.8	2.5	3.7	0.76
Girls	4.1	4.3	3.6	1.9	
**Governorate**	*p*^1^ = 0.069	*p*^1^ = 0.75	*p*^1^ = 0.23	*p*^1^ = 0.13	0.039
Capital	1.2	4.5	3.3	2.4	
Hawali	8.6	4.7	4.0	4.9	
Farwanyia	2.1	3.3	0.6	2.8	
Ahmadi	6.0	3.2	3.2	0.9	
Jahra	4.4	5.4	5.2	4.4	
Mubaral Al Kabeer	4.7	3.2	2.5	0.0	

^1^ *p* value for the percentages’ comparison by Chi-squared test across the period of study.

**Table 3:** By year, distribution of breakfast skipping, sugary and sweetened beverages weekly consumption and daily screen time use.

	2016	2017	2018	2019	*p* ^1^ Value (overall)
**N**	1184	1378	832	1006	
**Breakfast skipping**	22.2	19.3	20.4	19.9	<0.0001
**Total sugary and sweetened beverages**	5.92 ± 0.07 ^2^	5.85 ± 0.06	5.70 ± 0.08	5.62 ± 0.07	<0.0001
Carbonated drinks (times per week)	1.76 ± 0.06	0.96 ± 0.04	0.90 ± 0.05	0.97 ± 0.04	<0.0001
Non-fresh drinks (times per week)	4.16 ± 0.06	4.89 ± 0.05	4.80 ± 0.06	4.64 ± 0.05	<0.0001
**TV watching during the weekend**					
<2 h	76.2	78.4	79.1	85.2	<0.0001
2–3 h	17.5	14.3	13.8	11.2	
>3 h	6.3	7.3	6.5	3.6	
**TV watching during a weekday**					
<2 h	75.2	77.4	80.0	85.5	<0.0001
2–3 h	18.5	14.9	13.9	10.8	
>3 h	6.4	7.7	6.2	3.8	
**Computer playing during the weekend**					
<2 h	67.3	71.2	76.3	77.1	<0.0001
2–3 h	21.6	18.1	16.4	18.5	
>3 h	11.1	10.7	7.3	4.4	
**Computer use during a weekday**					
<2 h	67.3	70.5	76.8	79.4	<0.0001
2–3 h	21.8	18.8	16.3	16.8	
>3 h	10.9	10.7	6.9	3.8	

^1^ *p* value for the values comparison across the period of study (2016–2019) ^2^ mean ± standard error of mean.

**Table 4:** Logistic regression for the association of overweight and obesity with breakfast skipping, screen time and sugary and sweetened beverage consumption.

	Overweight		Obesity		BMI-for-Age z Score
	Crude OR (95% CI) ^1^	Adjusted ^2^ OR (95% CI) ^1^	Crude OR (95% CI) ^1^	Adjusted ^2^ OR (95% CI) ^1^	Coef. (95% CI)
**Breakfast skipping**	*p ^3^* = 0064	*p ^3^* = 0.0044	*p ^3^* = 0.75	*p ^3^* = 0.57	*p* = 0.14
No	1	1	1	1	1
Yes	0.69 (0.53–0.90)	0.69 (0.53–0.89)	0.93 (0.63–1.39)	0.88 (0.59–1.33)	−0.08 (−0.18–0.03)
**Daily TV watching**	*p* = 0.097	*p* = 0.13	*p* = 0.0007	*p* = 0.0006	*p* = 0.21
<2 h	1	1	1	1	1
2–3 h	1.30 (1.01–1.67)	2.02 (1.01–4.03)	2.05 (1.41–2.98)	5.63 (2.32–13.6)	0.29 (−0.04–0.63)
>3 h	0.91 (0.59–1.38)	1.10 (0.40–3.02)	1.45 (0.79–2.68)	1.17 (0.25–5.41)	−0.01 (−0.45–0.43)
**TV watching during a weekend**	*p* = 0.25	*p* = 0.34	*p* = 0.044	*p* = 0.021	*p* = 0.081
<2 h	1	1	1	1	1
2–3 h	1.21 (0.93–1.56)	0.61 (0.30–1.23)	1.61 (1.09–2.41)	0.26 (0.10–0.67)	−0.38 (−0.71–−0.05)
>3 h	0.88 (0.57–1.34)	0.67 (0.24–1.87)	1.40 (0.76–2.57)	0.86 (0.19–3.93)	−0.14 (−0.59–0.31)
**Daily computer use**	*p* = 0.40	*p* = 0.79	*p* = 0.0072	*p* = 0.78	*p* = 0.47
<2 h	1	1	1	1	1
2–3 h	1.15 (0.91–1.46)	0.81 (0.43–1.55)	1.68 (1.16–2.43)	1.07 (0.41–2.83)	−0.16 (−0.46–0.14)
>3 h	1.14 (0.82–1.60)	1.09 (0.42–2.84)	1.69 (1.02–2.79)	1.71 (0.38–3.93)	−0.19 (−0.64–0.24)
**Computer playing during the weekend**	*p* = 0.38	*p* = 0.82	*p* = 0.027	*p* = 0.96	*p* = 0.23
<2 h	1	1	1	1	1
2–3 h	1.16 (0.91–1.47)	1.22 (0.65–2.30)	1.55 (1.07–2.25)	1.05 (0.40–2.79)	0.25 (−0.04–0.55)
>3 h	1.14 (0.81–1.58)	1.16 (0.44–3.06)	1.59 (0.96–2.63)	0.84 (0.18–3.87)	0.17 (−0.27–0.61)
**Sugary and sweetened beverages (times per week)**	1.02 (0.98–1.06)	1.01 (0.97–1.05)	1.06 (0.99–1.14)	1.03 (0.96–1.11)	0.02 (0.003–0.04)

^1^ Odds ratio with 95% confidence interval. ^2^ Covariables: age; sex; breakfast status; daily use of TV; TV watching during the weekend; daily use of computer; computer playing during the weekend; sugary and sweetened beverage consumption; governorate. ^3^ Crude or adjusted *p*-value for association of overweight or obesity with co-factor.

## Data Availability

The data are the property of the Kuwait Ministry of Health.
